# Quantitative assessment of placental morphology may identify specific causes of stillbirth

**DOI:** 10.1186/s12907-016-0023-y

**Published:** 2016-02-09

**Authors:** Imogen Ptacek, Anna Smith, Ainslie Garrod, Sian Bullough, Nicola Bradley, Gauri Batra, Colin P. Sibley, Rebecca L. Jones, Paul Brownbill, Alexander E. P. Heazell

**Affiliations:** Institute of Human Development, Faculty of Medical and Human Sciences, University of Manchester, Oxford Rd, Manchester, M13 9PL UK; Maternal and Fetal Health Research Centre, 5th floor (Research), St Mary’s Hospital, Oxford Road, Manchester, M13 9WL UK; Department of Histopathology, Royal Manchester Children’s Hospital, Central Manchester University Hospitals NHS Foundation Trust, Manchester Academic Health Science Centre, Manchester, M13 9WL UK

**Keywords:** Stillbirth, Unexplained Stillbirth, Placental Morphometry, Fetal Growth Restriction, Villous vascularity, Avascular villi

## Abstract

**Background:**

Stillbirth is frequently the result of pathological processes involving the placenta. Understanding the significance of specific lesions is hindered by qualitative subjective evaluation. We hypothesised that quantitative assessment of placental morphology would identify alterations between different causes of stillbirth and that placental phenotype would be independent of post-mortem effects and differ between live births and stillbirths with the same condition.

**Methods:**

Placental tissue was obtained from stillbirths with an established cause of death, those of unknown cause and live births. Image analysis was used to quantify different facets of placental structure including: syncytial nuclear aggregates (SNAs), proliferative cells, blood vessels, leukocytes and trophoblast area. These analyses were then applied to placental tissue from live births and stillbirths associated with fetal growth restriction (FGR), and to placental lobules before and after perfusion of the maternal side of the placental circulation to model post-mortem effects.

**Results:**

Different causes of stillbirth, particularly FGR, cord accident and hypertension had altered placental morphology compared to healthy live births. FGR stillbirths had increased SNAs and trophoblast area and reduced proliferation and villous vascularity; 2 out of 10 stillbirths of unknown cause had similar placental morphology to FGR. Stillbirths with FGR had reduced vascularity, proliferation and trophoblast area compared to FGR live births. Ex vivo perfusion did not reproduce the morphological findings of stillbirth.

**Conclusion:**

These preliminary data suggest that addition of quantitative assessment of placental morphology may distinguish between different causes of stillbirth; these changes do not appear to be due to post-mortem effects. Applying quantitative assessment in addition to qualitative assessment might reduce the proportion of unexplained stillbirths.

**Electronic supplementary material:**

The online version of this article (doi:10.1186/s12907-016-0023-y) contains supplementary material, which is available to authorized users.

## Background

Histological examination of the placenta is one of the most frequently performed investigations to identify the cause of death in cases of stillbirth [1]; its application in this context is recommended by international guidelines [2–4]. A recent systematic review found large variations in the methodological quality of studies of placental examination after stillbirth, with few studies of high quality [5]. Interpretation of the results of such studies is further complicated by the use of different classification systems and a lack of consensus in terminology used to describe placental lesions which results in a large variation in the proportion of stillbirths attributed to a placental “cause” from 11–65 % [5]. Such qualitative placental assessment, combined with varied terminology, has some deficiencies. Firstly, qualitative assessment of placental lesions may introduce bias, particularly if assessors are not blinded to outcome. Furthermore, qualitative assessment may lead to inter-observer variation in diagnoses, which ranged from 25–91 % in one study [6]. The significance of specific abnormalities to stillbirth has also been questioned by Pathak et al. who describe placental abnormalities in a significant proportion of apparently healthy live-born infants [7]. Finally, identification of a specific lesion does not imply a single cause. For example, appearances of fetal thrombotic vasculopathy have been associated with various pathologies including: cytomegalovirus infection [8], umbilical cord accidents [9] or specific patterns of umbilical cord coiling [10]. Similarly, changes of maternal underperfusion may be related to hypertensive disorders [11] and antiphospholipid syndrome [12].

Recently, significant advances have been made in the development of modern classification systems [13–15] that reduce the proportion of unexplained stillbirths [16]. These classification systems have given greater recognition to the role of placental pathology in the aetiology of stillbirth [13–15] and progress has been made in reducing the variation of placental histological findings [6]. However, these clinically-orientated descriptions of placental phenotype have not yet been adopted into widespread practice, in part due to continued debate about terminology which varies between clinical and research studies [17]. Research studies have employed quantitative descriptions of placental morphology by stereology and morphometry to describe differences in placental structure in clinical conditions related to stillbirth such as fetal growth restriction (FGR) [18] and reduced fetal movements [19]. We aimed to use these quantitative methods to objectively evaluate placental appearances of different causes of stillbirth. Firstly, we hypothesised that specific causes of stillbirth would be associated with a morphometric phenotype. Secondly, we hypothesised that morphological abnormalities associated with stillbirth would differ from live births with the same condition. To be of diagnostic value, any observed changes should not represent artefacts of storage or cessation of fetal blood flow after death. Since we have already described the effects of placental storage on placental structure [20], here we address a third hypothesis that there is an acute effect of post-stillbirth fetoplacental haemostasis on placental morphology.

## Methods

### Placental tissue samples

To address the first hypothesis we obtained placental tissue from cases of stillbirth, defined as the birth of an infant with no signs of life after 24 weeks gestation. Parents gave permission for the use of samples for research at the time of consent for post-mortem examination. A favourable ethical opinion was given by the Greater Manchester South Research Ethics Committee (09/H1012/11) and approval given from the Research and Innovation Division of Central Manchester University Hospitals NHS Foundation Trust to conduct the study. Cases of stillbirth were classified using the ReCoDe system [15] by a multidisciplinary meeting following a full panel of investigations including: post-mortem, histopathological examination of the placenta, chromosomal analysis and maternal biochemical, haematological, immunological and serological tests. We obtained samples from the following classifications of stillbirth: cord accident (*n* = 8), diabetes (*n* = 5), FGR (*n* = 10), hypertension (*n* = 8), infection (*n* = 8) and from stillbirths of an unknown cause (*n* = 10). For comparison, matched placental samples were used from appropriately-grown live born infants and preterm births (26-36 weeks) (demographics shown in Table 1). Samples from live births were collected following written informed consent as part of the Maternal and Fetal Research Centre (MFHRC) Biobank (08/H1010/55). To address the second hypothesis, placental samples were taken from a further cohort of stillbirths attributed to FGR (*n* = 13) and from live births with FGR (*n* = 13) with the same ethical approvals as described above. FGR was defined as a customised birthweight <5th centile (demographics shown in Table 2). To address the final hypothesis, placental tissue was obtained from appropriately grown (*n* = 7) and FGR (*n* = 5) live births following written informed consent as part of the MFHRC biobank previously described. For all cases, maternal and infant demographic information was recorded from medical case notes and post-mortem reports (for stillbirths). An estimate of the duration of in utero retention was made according to Genests’ descriptions of findings at post-mortem and from histopathological examination of the placenta [21–23].

**Table 1:** Demographic characteristics of samples from live births and stillbirths from known and unknown causes. Birthweight was significantly lower in stillbirths from FGR and hypertension than live births (*P* < 0.01); all other variables did not significantly differ between groups. Data are presented as median with range in parentheses except for estimated time of retention in utero where number of cases are presented

		Live births	Preterm birth	Cord	Diabetes	Hypertension	Infection	FGR	Unknown
Number of Samples		10	7	8	5	8	9	10	10
Maternal Age (years)		32 (28–37)	27 (18–41)	28 (21–36)	34 (31–40)	33 (27–37)	30 (25–32)	30 (22–32)	28 (21–31)
Gravidity		1 (1–5)	3 (1–6)	1 (1–1)	4 (2–7)	1 (1–2)	1 (1–3)	1 (1–4)	1 (1–2)
Parity		0 (0–4)	0 (0–4)	0 (0–0)	1 (1–2)	0 (0–0)	0 (0–2)	0 (0–2)	0 (0–0)
Gestation at delivery (weeks)		37 (37–38)	31 (26–36)	30 (27–38)	28 (28–40)	31 (26–35)	38 (24–41)	34 (26–38)	31 (27–39)
Birthweight (g)		3090 (2805–3430)	1821 (786–2760)	1300 (702–2750)	3120 (1473–3590)	1110 (399–1730)	2680 (494–2985)	1065 (564–2230)	2170 (892–3150)
Estimated in utero retention time	0 h - <24 h	N/A	N/A	0	1	4	4	2	2
	≥24 h - < 48 h			1	0	2	2	4	3
	≥48 h - < 96 h			2	1	0	2	2	1
	≥96 h - <1 week			3	1	1	0	0	2
	≥1 week			2	2	1	1	2	2

**Table 2:** Demographic details of samples used for experimental comparisons. The left hand columns relate to samples obtained from cases of FGR that were live or stillborn; all these samples had a birthweight <5th centile. The right hand columns relate to samples used for perfusion experiments. Data are presented as median with range in parentheses

	FGR live birth	FGR still birth	Appropriate for gestational age - placental perfusion	FGR - placental perfusion
Number of Samples	13	13	7	5
Maternal Age (years)	32 (20–40)	23 (19–42)	33 (27–36	31 (23–39))
Gravidity	2 (1–9)	2 (1–3)	2 (2–4)	1 (1–5)
Parity	1 (0–8)	0 (0–2)	2 (1–2)	1 (0–5)
Gestation at delivery (weeks)	37 (30–41)	35 (28–38)	39 (39–39)	38 (35–39)
Birthweight (g)	1389 (536–3060)	1010 (385–2000)	3740 (2910–3940)	2262 (1900–2440)
Mode of delivery (% Caesarean)	69	0	71	89
Infant gender (% Female)	54	62	57	78

Tissue from live births was obtained within 30 min of delivery. For assessment of placental morphology biopsies of villous tissue were dissected from the centre, middle and edge of the placenta. Tissue was fixed in 4 % neutral buffered formalin for 24 h before being wax embedded. For stillbirth samples three blocks of placental tissue not obtained from specific lesions were obtained for each placenta.

### Placental perfusion

Unless otherwise stated, all reagents were supplied by Sigma-Aldrich Chemical Company (Poole, UK). To examine the acute impact of continued maternal blood flow in the absence of fetal blood flow single-sided (maternal) ex vivo human placental lobule perfusion was adapted from the dual-sided perfusion model [24]. Perfusion was performed on placentas from normal pregnancy (*n* = 7) and placentas from pregnancies complicated by FGR (*n* = 5). An intact peripheral lobule was selected, devoid of post-partum tears, deep decidual damage and marginal membrane separations. Prior to perfusion two villous biopsies were sampled from neighbouring lobules taken 5 cm apart, and fixed immediately in 4 % neutral buffered formalin forming “pre-perfusion samples”. The fetal artery and vein on the chorionic surface, serving the villous trees within the lobule designated for perfusion, were each ligated using sutures (Mersilk 3/0, Ethicon, supplied by NuCare, UK) to confine a static fetal blood pool within the fetal vasculature of the associated cotyledons. The maternal surface was cannulated using five 10 cm lengths of polythene tubing (Smiths Medical, UK) arising from a perfusion manifold (Harvard Apparatus, UK). The distal ends of the cannulae were cut into apices and inserted through the decidual surface of the lobule with an even spatial distribution. The perfusate was modified Earle's bicarbonate buffer (EBB 117 mM NaCl, 10.7 mM KCl, 5.6 mM D-glucose, 3.6 mM CaCl, 1.8 mM NaH_2_PO_4_, 13.6 mM NaHCO_3_, 0.04 mM L-arginine, 0.8 mM MgSO_4_, 3.5 % (w/v) dextran, 0.1 % (w/v) bovine serum albumin, 5000 IU/L Heparin sodium) equilibrated with 95 % O_2_ / 5 % CO_2_ to pH 7.4 and warmed to 37 °C, delivered by a roller pump (Watson Marlow, UK) at 14 ml/min. Lobule preparations were only considered acceptable for experimentation when maternal-side perfusion was established within 30 min of delivery. Open-circuit perfusion was for 6 h, and then the physiological buffer was switched to a 4 % neutral buffered formalin at T = 6 h for a 10 min maternal-side perfusion fixation period. Following this, the lobule was excised and two further full thickness (vertical and horizontal) biopsies slices were taken as the “post-perfusion samples”. These wide tissue sections where then immersion fixed in 4 % neutral buffered formalin for 24 h before being wax embedded. Placental structure in these biopsies was examined as described above.

### Immunohistochemistry

Placental cell turnover, structure and vascularity were assessed using antibodies specific for Ki67 (Dako, Ely, Cambridgeshire, UK; 0.16 μg/ml), cytokeratin 7 (Dako; 0.9 μg/ml) and CD31 (Dako; 0.16 μg/ml). The number of leukocytes was assessed by an antibody specific for CD45 (Dako; 0.4 μg/ml). Negative controls were performed using non-immune mouse IgG (Dako) at matching concentrations to the primary antibody. Immunohistochemistry was performed as previously described with antigen retrieval performed by microwaving the sections for 10 min in 0.01 M sodium citrate buffer [19, 20].

Quantification of syncytial nuclear aggregates (SNAs, also known as syncytial knots) was conducted on sections stained with haematoxylin and eosin as previously described [19, 20]. Dewaxed and rehydrated sections were stained with Harris’s haematoxylin for 10 min before differentiation in acid-alcohol. Slides were stained with eosin for 2 min, rinsed in cold tap water, and dehydrated and mounted as described above.

For all analyses images were captured using an Olympus BX41 light microscope (Southend-on-Sea, UK) and QIcam Fast 1394 (QImaging, BC, Canada) and Image Pro Plus 6.0 and 7.0 (Media Cybernetics Inc., MD, USA). During image acquisition and analysis the presumed cause of stillbirth was concealed from the observer. Between images the microscope was taken out of focus to prevent selection bias, if the randomly selected image was not mostly of terminal villi another area was identified. Five random images of terminal villi were taken of each section, giving a total of 15 images per placenta for each component evaluated.

### Assessment of placental structure

The number of SNAs were counted and total villous area measured using image analysis software, expressed as the number of SNAs per mm^2^ of villous tissue as previously described [25]. Proliferative index was the number of Ki67 positive nuclei as a proportion of total nuclei as previously described [19, 20]. Vascularity was expressed as the number of capillaries per terminal villus and the percentage of avascular villi (defined as a villus with no evidence of CD-31 immunostaining or morphological evidence of vessels) [19, 20]. Trophoblast area was expressed as the proportion of villous area positive for CK-7 immunostaining. The number of leukocytes was assessed by the number of CD45 positive cells per 1,000 nuclei.

### Statistical analysis

For comparison of different causes of stillbirth data from each variable was compared to the median level in healthy controls using Wilcoxon signed rank test. Cases of FGR that were live-born were compared to those who were stillborn using Mann-Whitney *U* test. Data from pre- and post-perfusion samples were compared using Wilcoxon matched-pairs test. Demographic variables were compared using Kruskal-Wallis test with Dunn’s post-hoc test for multiple comparisons and Mann-Whitney *U* test for single comparisons. For all statistical tests a p-value of 0.05 was considered to be statistically significant. All statistical analyses were carried out using GraphPad PRISM (Version 6, La Jolla, CA).

## Results

### Placental morphology in different causes of stillbirth

**Fig. 1:**
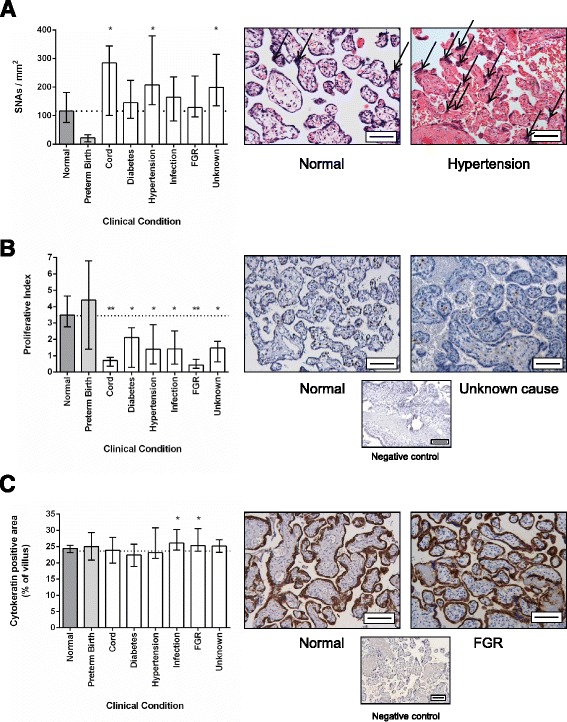
**a** Assessment of syncytial nuclear aggregates (SNAs) in different causes of stillbirth compared to healthy live births. SNAs are shown by open arrows in representative images from normal pregnancy and stillbirth associated with hypertension. **b** Assessment of proliferation in different causes of stillbirth compared to healthy live births. **c** Assessment of trophoblast area in different causes of stillbirth compared to healthy live births. Negative control images shown in small panel beneath representative images of normal pregnancy and FGR. Scale bar = 50 μm in all images. Graphs show median and range, * *p* < 0.05, ** *p* < 0.01, *** *p* < 0.001. Dotted line indicates median level of healthy control

**Fig. 2:**
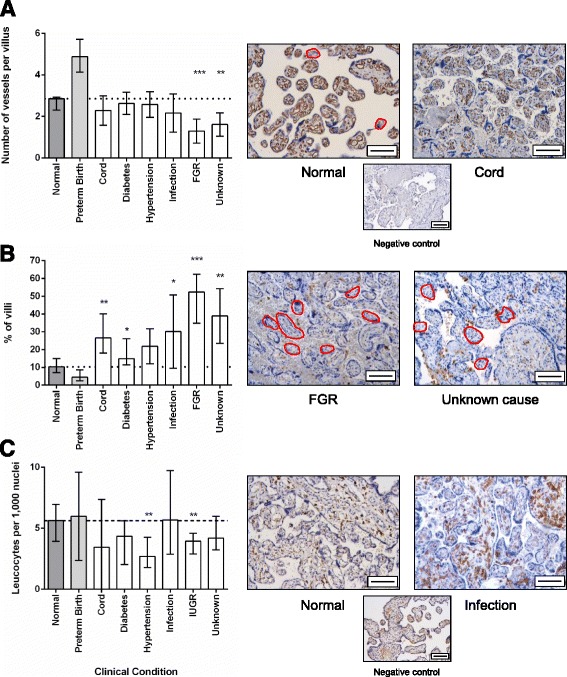
**a** Assessment of villous vascularity in different causes of stillbirth compared to healthy live births. **b** Proportion of avascular villi in different causes of stillbirth compared to healthy live births. Avascular villi are highlighted in red. **c** Assessment of the number of leukocytes in different causes of stillbirth compared to healthy live births. Negative control images shown in small panel beneath representative images of normal pregnancy and FGR. Scale bar = 50 μm in all images. Graphs show median and range, * *p* < 0.05, ** *p* < 0.01, *** *p* < 0.001. Dotted line indicates median level of healthy control

In comparison to normal pregnancy, SNAs were increased in stillbirths attributed to cord accident, hypertension, FGR and in stillbirths with an unknown aetiology (Fig. 1a). This was in contrast to fewer SNAs seen in preterm live births. Proliferation was reduced in all cases of stillbirth, but was particularly reduced in those cases attributed to cord accident or FGR (Fig. 1b). The median trophoblast area (measured as cytokeratin-7 positive area) was increased in stillbirths attributed to infection and FGR (Fig. 1c). The median number of blood vessels identified by CD31 immunostaining was significantly reduced in stillbirths attributed to FGR and those with an unknown cause (Fig. 2a). The number of avascular villi was significantly increased in these conditions, although an increase in avascular villi was also seen in stillbirths attributed to cord compression and hypertension (Fig. 2b). These changes were in contrast to an increase in vascularity and reduction in avascular villi observed in preterm live births. The median number of leukocytes was reduced in stillbirths attributed to maternal hypertension and FGR compared to healthy controls (Fig. 2c). It is important to note that in some variables, notably the number of leukocytes in cases of infection, there was a wide range in measurements obtained. The cause of stillbirth with the most placental differences from healthy pregnancies was FGR, which has increased numbers of SNAs, reduced proliferation, increased trophoblast area, fewer blood vessels per villus, more avascular villi and decreased numbers of leukocytes. Interestingly, the condition with next most frequent abnormalities was stillbirths of unknown cause. When the individual profiles of stillbirths from unknown cause are examined, two had a similar profile to those with FGR and others had similar features such as increased density of SNAs and reduced vascularity (Table 3). None of the features examined altered according to the estimated duration of in utero retention (Additional file 1: Figure S1).

**Table 3:** Pattern of placental morphology in placental samples from stillbirths of unknown cause (*n* = 10) demonstrating two samples with a very similar pattern to samples from FGR (highlighted in grey)

Sample	SNAs	Proliferation	Vascularity	Avascular villi	Trophoblast	Leukocytes	Profile
Unknown 1	Low	High	Unchanged	High	Low	Low	Not similar
Unknown 2	High	Unchanged	Low	High	Unchanged	Unchanged	Not similar
Unknown 3	High	Low	Low	High	High	Unchanged	Similar to FGR
Unknown 4	High	Unchanged	High	Unchanged	Unchanged	Unchanged	Not similar
Unknown 5	High	Low	Low	High	High	Unchanged	Similar to FGR
Unknown 6	High	Unchanged	Low	High	Unchanged	Unchanged	Not similar
Unknown 7	Unchanged	Unchanged	Low	High	Unchanged	Unchanged	Not similar
Unknown 8	Unchanged	High	Low	High	Increased	Low	Not similar
Unknown 9	High	Unchanged	Low	High	Increased	High	Not similar
Unknown 10	Unchanged	Unchanged	Low	High	Increased	Unchanged	Not similar

**Fig. 3:**
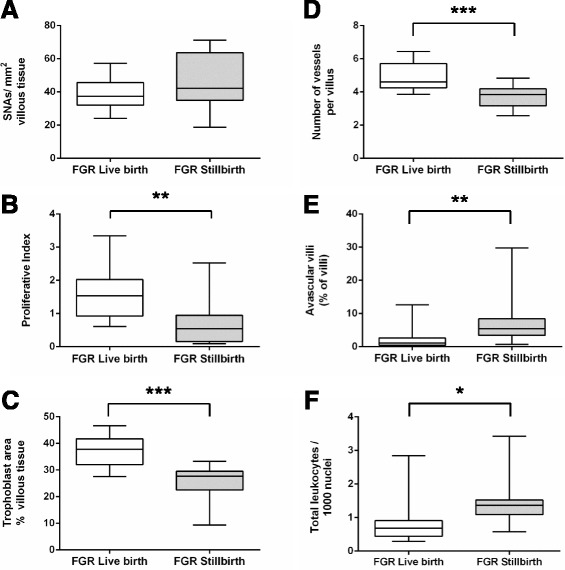
Assessment of placental morphometry in live births associated with FGR compared to stillbirths associated with FGR. Graphs present data for **a**) Syncytial nuclear aggregates (SNAs) **b**) Proliferation, **c**) Trophoblast area, **d**) Villous vascularity, **e**) Proportion of avascular villi and **f**) Number of Leukocytes. Graphs show median and range, * *p* < 0.05, ** *p* < 0.01, *** *p* < 0.001

When compared to FGR live births, FGR stillbirths did not have increased numbers of SNAs but had reduced proliferation and trophoblast area, fewer blood vessels per villus and a greater proportion of avascular villi. Leukocytes were increased in FGR stillbirths compared to FGR live births (Fig. 3).

### Effect of short-term fetoplacental haemostasis on placental morphology

**Fig. 4:**
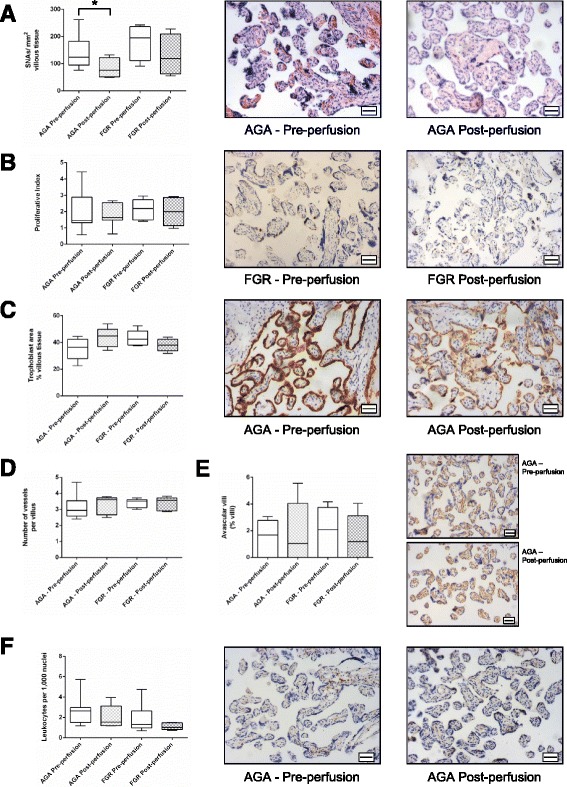
Assessment of placental morphometry before and after maternal-side only placental perfusion in normal and FGR placentas. Graphs present data for a reduction in **a**) syncytial nuclear aggregates (SNAs), but no change in **b**) Proliferation, **c**) Trophoblast area, **d**) Villous vascularity, **e**) Proportion of avascular villi and **f**) Number of Leukocytes. Graphs show median and range, * *p* < 0.05. Representative images of each feature are shown. Scale bar = 50 μm in all images

To assess changes that may happen in utero after cessation of fetal blood flow, placental tissue was examined before and after maternal-side only placental perfusion in placental tissue from healthy and FGR pregnancies. Perfusion in this manner for 6 h was not associated with any changes in proliferation, trophoblast area, villous vascularity or the proportion of avascular villi (Fig. 4). Maternal-side only perfusion was associated with a reduction in the number of SNAs in normal tissue (Fig. 4a). There was a consistent trend towards lower numbers of leukocytes in perfused tissue from both appropriately-grown and FGR pregnancies (*p* = 0.08).

## Discussion

This pilot study demonstrates that objective assessment of placental morphology may provide additional information on placental villous structure in cases of stillbirth and in some cases, such as in FGR, can differentiate between specific causes of stillbirth and healthy live-born infants. In other cases, such as stillbirths attributed to maternal diabetes, there were no morphological differences from live-born infants, which is consistent with few histopathological abnormalities in stillbirths related to diabetes [26]. When the morphometric profile was applied to ten stillbirths of unknown cause, two had a very similar placental profile to FGR, which suggests that some stillbirths that currently have no identified cause (despite intensive investigation) may actually result from FGR in a fetus that was not small, i.e. infants who have a birthweight >10th centile but whose growth rate was slowing down. These preliminary findings suggest that addition of objective assessment of placental morphology to histological examination with the use of a modern classification system may further decrease the proportion of unexplained stillbirths. However, further research is needed to understand the role that abnormalities of placental structure and function have in the aetiology of stillbirth both in the presence of a small fetus and when the birthweight is within an accepted normal range [27].

The findings in FGR stillbirths are consistent with other stereological and morphometric assessment of FGR placentas including: increased SNAs [25], reduced villous vascularity [28], reduced proliferation [29] and number of cytotrophoblasts [28]. The pattern seen in FGR stillbirths was also consistent with the placental morphology in women with reduced fetal movements [19], who are at increased risk of stillbirth [30]. However, some results differ from other studies, one of which describes a positive relationship between trophoblast area and birthweight [31]. The reduced trophoblast area in stillbirth FGR compared to healthy live births contrasts with an increased area relative to live born FGR infants. This observation may result from thicker syncytiotrophoblast covering hypoplastic villi; application of stereological techniques is required to explore this observation in greater depth.

The findings of this study are consistent with the Stillbirth Collaborative Research Network (SCRN) case-control study which found increased presence of placental lesions in stillbirths, including: diffuse terminal villous immaturity, inflammation, vascular degeneration in the chorionic plate, intra-placental thrombi, avascular villi and parenchymal infarction [32]. The SCRN study found differences in lesions depending on gestation. Avascular villi and fetal vascular thrombi were more frequently seen in term stillbirths than those occurring at earlier gestations. Whereas, chorioamnionitis was seen less frequently in stillbirths than live births at 24 weeks’ gestation, but more frequently in term stillbirths compared to matched live births [32]. The SCRN study provides evidence that different causes of stillbirth have a different placental phenotype and addition evidence that gestation may affect the cause of stillbirth. Our study demonstrated that some features (SNAs and villous vascularity) were altered in preterm compared to term live births. Critically, these changes were in the opposite direction to that seen in stillbirth, so the morphology changes seen in cases of stillbirth cannot be attributed to their preterm gestation.

The finding that the placental phenotype of FGR stillbirths had reduced villous vascularity, increased avascular villi and increased leukocyte infiltration compared to live-born FGR may imply that FGR stillbirths result from a more severe placental phenotype. However, these differences must be interpreted cautiously, as differences between live and stillbirth may also result from artefacts from cessation of fetoplacental blood flow after fetal death, differences in mode of delivery or from storage prior to fixation. Our previous experimental data suggest that storage prior to fixation for ≤48 h does not alter any of the indices measured here [20]. Studying the effects of in utero retention is more challenging. We attempted to model this by maternal-side only placental perfusion for 6 h, finding that this did not reproduce any of the differences between FGR stillbirths and live births. However, the duration of perfusion was limited by the experimental technique and it cannot reproduce the in utero environment (e.g. presence of the maternal immune system). Placental changes may be altered by the duration of in utero retention, as evident by changes in histopathological appearances of the placenta in fetal maceration [33]. Thus, further study is needed to determine the effects of potential confounders, particularly in utero retention, on the quantitative measures used in this study. This could be explored by evaluating the morphology of stillbirths with known in utero retention time (e.g. intrapartum events, feticide for structural anomaly). The possibility that morphological changes might arise from differences in mode of delivery should also be considered, as Caesarean section is rarely used in cases of stillbirth, but is frequently employed in live born FGR infants; this can be resolved by detailed study of placental morphology after vaginal delivery and Caesarean section.

The study reported here is strengthened by a detailed assessment of multiple aspects of placental morphology with blinding of assessor to study group or pregnancy outcome. We also have compared cases of stillbirth to appropriately-grown healthy controls and preterm births primarily recruited for research rather than clinical cases with indication(s) for perinatal histology. The use of objective techniques that also have been used to evaluate related conditions allows comparison between different clinical situations. However, this study does have limitations: although 50 samples from well-characterised stillbirths have been analysed, this only amounts to ≤10 samples per group and these were obtained from a single Paediatric and Perinatal Pathology department. Unfortunately, at the time of collection the collection protocols between the clinical histopathological service and research laboratories were slightly different resulting in different numbers of samples obtained per placenta potentially introducing a bias between samples from stillbirths and live births. However, we believe the chance of selection bias to be low as placental tissue was randomly sampled and blocks of specific lesions were not used in either protocol. Samples were divided into groups based upon the classification of stillbirth determined by multidisciplinary review (involving obstetricians, midwives, sonographers and pathologists) and, although this was made as robust as possible, it is possible that the cause of death was different from that attributed in the perinatal review process.

## Conclusion

Due to the critical role played by the placenta in determining the outcome of pregnancy and the role of placenta failure in the aetiology of stillbirth [34], placental histology is a frequently employed investigation that can provide important information for clinicians and parents regarding the reasons for their child’s death [35]. When combined with a modern classification system, histological examination of the placenta reduces the proportion of unexplained stillbirths [36, 37]. Our preliminary findings suggest that addition of objective measurement of placental structure may add to understanding of the cause of stillbirth. These quantitative observations need to be related to established qualitative descriptions; in some cases such as avascular villi and fetal thrombotic vasculopathy this may be straightforward, in others, such as placental maturation disorders, this may be more complex. Prior to clinical application further studies are needed to develop normal ranges for these morphological characteristics at different gestational ages and to determine the effects of in utero retention on these indices. Then blinded studies of randomly sampled cases of stillbirth from multiple populations are needed to ensure the findings presented here are sufficiently sensitive and specific for diagnostic use.

## Additional file

Additional file 1: Figure S1. Assessment of placental morphometry in stillbirths (irrespective of cause) grouped by estimated time of in utero retention according to Genest’s criteria [21–23]. Graphs present data for A) Syncytial nuclear aggregates (SNAs) B) Proliferation, C) Trophoblast area, D) Villous vascularity, E) Proportion of avascular villi and F) Number of Leukocytes per 1,000 nuclei. Graphs present the median and interquartile range for each group. There is no statistically significant difference of the frequency of the morphological feature and the groups divided by in utero retention. (PPTX 197 kb)

## Abbreviations

EBB: Earles’ Bicarbonate Buffer
FGR: Fetal growth restriction
MFHRC: Maternal and Fetal Health Research Centre
ReCoDe: Relevant Condition at Death (Classification System)
SCRN: Stillbirth Collaborative Research Network
SNA: Syncytial nuclear aggregate
