# Supplementary figures and images for: Quantitative assessment of placental morphology may identify specific causes of stillbirth
Source: BMC Clin Pathol. 2016 Feb 9;16:1. doi: 10.1186/s12907-016-0023-y (PMC4748636; doi:10.1186/s12907-016-0023-y)

## Slide 1
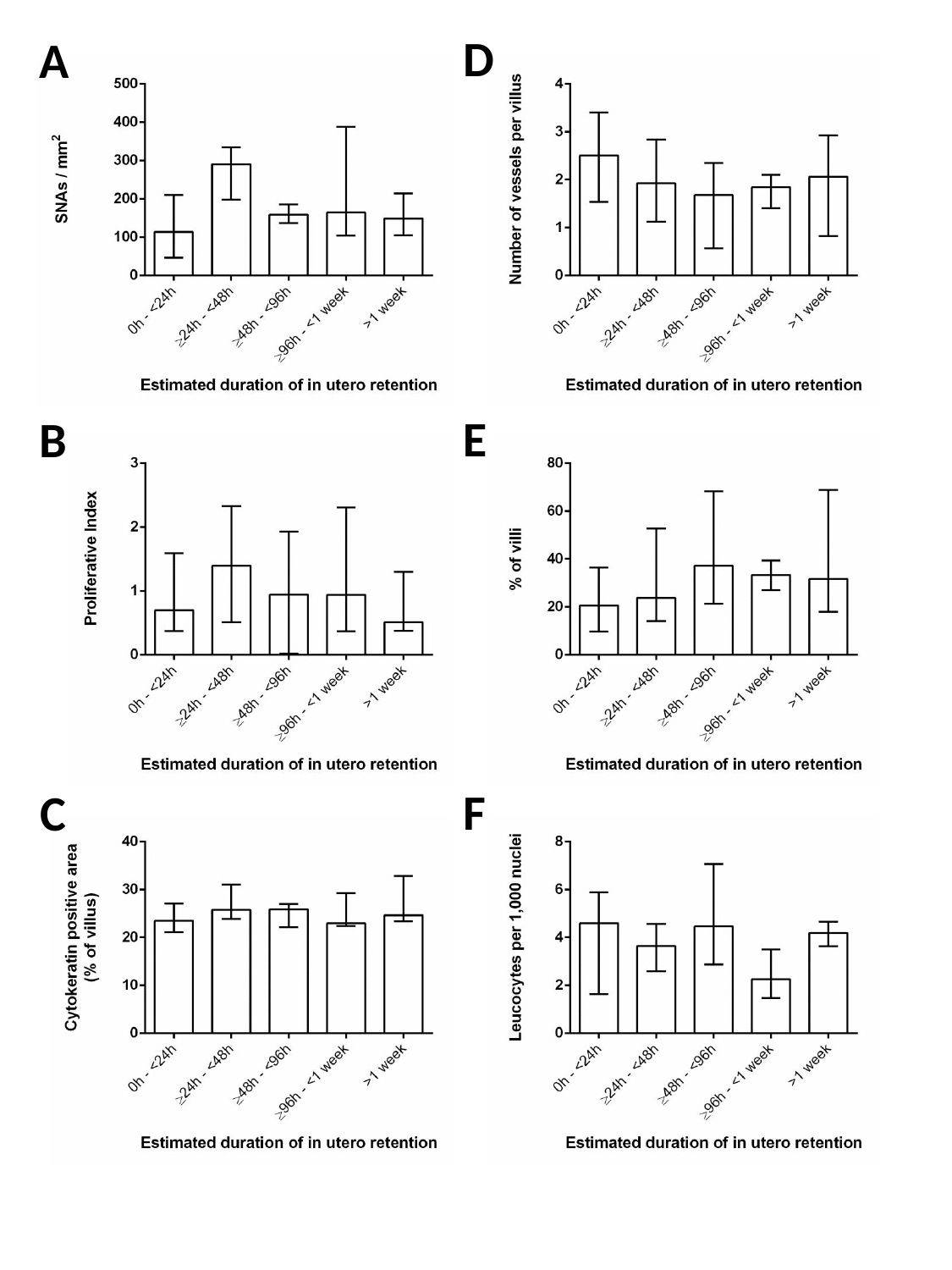

D
A
E
B
F
C

Supplement: Additional file 1: Figure S1. — Assessment of placental morphometry in stillbirths (irrespective of cause) grouped by estimated time of in utero retention according to Genest’s criteria [21–23]. Graphs present data for A) Syncytial nuclear aggregates (SNAs) B) Proliferation, C) Trophoblast area, D) Villous vascularity, E) Proportion of avascular villi and F) Number of Leukocytes per 1,000 nuclei. Graphs present the median and interquartile range for each group. There is no statistically significant difference of the frequency of the morphological feature and the groups divided by in utero retention. (PPTX 197 kb) [file 12907_2016_23_MOESM1_ESM.pptx]
